# Updating the evidence for the role of corticosteroids in severe sepsis and septic shock: a Bayesian meta-analytic perspective

**DOI:** 10.1186/cc9182

**Published:** 2010-07-13

**Authors:** John L Moran, Petra L Graham, Sue Rockliff, Andrew D Bersten

**Affiliations:** 1 Department of Intensive Care Medicine, The Queen Elizabeth Hospital, 28 Woodville Road, Woodville, South Australia 5011, Australia; 2 Department of Statistics, Faculty of Science, Macquarie University, Balaclava Road, North Ryde, NSW 2109, Australia; 3 Department of Library Services, The Queen Elizabeth Hospital, 28 Woodville Road, Woodville, South Australia 5011, Australia; 4 Department of Critical Care Medicine, Flinders Medical Centre and School of Medicine, Flinders University, Sturt Road, Bedford Park, South Australia 5042, Australia

## Abstract

**Introduction:**

Current low (stress) dose corticosteroid regimens may have therapeutic advantage in severe sepsis and septic shock despite conflicting results from two landmark randomised controlled trials (RCT). We systematically reviewed the efficacy of corticosteroid therapy in severe sepsis and septic shock.

**Methods:**

RCTs were identified (1950-September 2008) by multiple data-base electronic search (MEDLINE via OVID, OVID PreMedline, OVID Embase, Cochrane Central Register of Controlled trials, Cochrane database of systematic reviews, Health Technology Assessment Database and Database of Abstracts of Reviews of Effects) and hand search of references, reviews and scientific society proceedings. Three investigators independently assessed trial inclusion and data extraction into standardised forms; differences resolved by consensus.

**Results:**

Corticosteroid efficacy, compared with control, for hospital-mortality, proportion of patients experiencing shock-resolution, and infective and non-infective complications was assessed using Bayesian random-effects models; expressed as odds ratio (OR, (95% credible-interval)). Bayesian outcome probabilities were calculated as the probability (*P*) that OR ≥1. Fourteen RCTs were identified. High-dose (>1000 mg hydrocortisone (equivalent) per day) corticosteroid trials were associated with a null (*n *= 5; OR 0.91(0.31-1.25)) or higher (*n *= 4, OR 1.46(0.73-2.16), outlier excluded) mortality probability (*P *= 42.0% and 89.3%, respectively). Low-dose trials (<1000 mg hydrocortisone per day) were associated with a lower (*n *= 9, OR 0.80(0.40-1.39); *n *= 8 OR 0.71(0.37-1.10), outlier excluded) mortality probability (20.4% and 5.8%, respectively). OR for shock-resolution was increased in the low dose trials (*n *= 7; OR 1.20(1.07-4.55); *P *= 98.2%). Patient responsiveness to corticotrophin stimulation was non-determinant. A high probability of risk-related treatment efficacy (decrease in log-odds mortality with increased control arm risk) was identified by metaregression in the low dose trials (*n *= 9, slope coefficient -0.49(-1.14, 0.27); *P *= 92.2%). Odds of complications were not increased with corticosteroids.

**Conclusions:**

Although a null effect for mortality treatment efficacy of low dose corticosteroid therapy in severe sepsis and septic shock was not excluded, there remained a high probability of treatment efficacy, more so with outlier exclusion. Similarly, although a null effect was not excluded, advantageous effects of low dose steroids had a high probability of dependence upon patient underlying risk. Low dose steroid efficacy was not demonstrated in corticotrophin non-responders. Further large-scale trials appear mandated.

## Introduction

In 1974, Weitzman and Berger reviewed the clinical trial design of studies reporting corticosteroid use in bacterial infections over the previous 20 years because of the controversial role of the therapeutic use of corticosteroids in acute infections [1]. It is ironic that 34 years later a similar sentiment was echoed: "For more than five decades, no other adjunctive therapy has been more consistently debated than the use of corticosteroids for severe sepsis and septic shock" [2]. A contemporaneous review concluded that the role of glucocorticoid therapy in intensive care remained uncertain [3]. In 1995, two meta-analyses found no benefit for high-(pharmacological)-dose corticosteroids in sepsis and septic shock [4,5] and in 2004 another two meta-analyses [6,7] found benefit for long courses of low-(stress) [7]-dose corticosteroids. This benefit was either qualified: pending the results of the Corticosteroid Therapy of Septic Shock (CORTICUS) [8] study, clinical equipoise remained for the issues of adreno-corticotrophin (ACTH) administration, cortisol testing, and the therapeutic use of hydrocortisone [9]; or more definitive: '...a beneficial therapy to critically ill patients in septic shock' [10]. That the confirmatory [11] phase III CORTICUS study [8] was 'somewhat disappointing' [12] undoubtedly reflects this history of therapeutic uncertainty. Current guidelines advocate a role for intravenous hydrocortisone in adult septic shock patients who are poorly responsive to fluid and vasopressor therapy and, in the apparent absence of a mortality effect dependent on ACTH responsiveness, attention has been directed to the more rapid time-resolution of shock with corticosteroids [13,14].

Thus the question still remains: what is the evidence, post CORTICUS [8], for the efficacy of corticosteroids in severe sepsis and septic shock? We undertook a systematic review and quantitative analysis of randomized controlled trials (RCT) addressing corticosteroid efficacy in severe sepsis and septic shock, updating [15] previous studies [6,7]. As the question of further large-scale trials to assess corticosteroids in septic shock has currently been canvassed [16], in particular the efficacy at high mortality risk [12], we addressed the risk-related efficacy of steroids within the trials considered [17] and estimated the predictive distribution for the underlying effect in new studies [18].

## Materials and methods

### Trial selection

Randomised controlled trials in critically ill patients evaluating corticosteroid therapy versus no corticosteroid therapy in severe sepsis or septic shock were considered for inclusion. Only trials reporting mortality were included. We excluded: studies reporting only physiological endpoints (for example, changes in immunological variables); descriptive studies; retrospective cohort studies; studies in the pediatric population; and studies exclusively reporting series of meningitis, typhoid fever and pneumonia where sub-set analyses of patients of interest (for this meta-analysis) were not reported. Where there was missing data or ambiguity of data presentation, attempts were made to contact the study author(s) to resolve these issues.

### Search strategy and quality assessment

An extensive computerized literature search was performed (SR) for the period of 1950 to September 2008 using the National Library of Medicine MEDLINE via OVID, OVID PreMedline, EBSCO Cinahl, OVID Embase, Cochrane Central Register of Controlled trials, Cochrane database of systematic reviews, American College of Physicians Journal Club, Health Technology Assessment Database and Database of Abstracts of Reviews of Effects. We restricted the search to studies on adult human populations and used the Mesh, Embase and Cinahl thesaurus in addition to free text searching. The following terms were identified as the most relevant: sepsis or bacteremia or fungemia or pneumonia or septicemia or septic shock narrowed down with the terms hydrocortisone or corticosteroids or adrenal cortex hormones or steroids. The set was then further limited to randomised controlled trials or clinical trials or multicenter study and trials published in English. A detailed search strategy is provided in Additional file 1.

We reviewed the abstracts of trials generated by the electronic search and the full text of trials pertaining to corticosteroids in sepsis and septic shock were retrieved for a more detailed evaluation. Review articles were examined to identify additional trials. In addition a hand search of the proceedings of scientific meetings of the following journals was performed: *American Journal of Respiratory and Critical Care Medicine*, *Chest*, *Critical Care Medicine*, *European Respiratory Journal*, *Intensive Care Medicine *and *Thorax*.

### Quality assessment

Three investigators (JLM, PLG, and AB) reviewed studies fulfilling inclusion criteria and pre-defined variables and outcomes were abstracted into standardized data abstraction forms. Quality assessment on the published studies was performed in an un-blinded fashion by two investigators (JLM, PLG) using the 11-point quality score of Cronin and colleagues [4]. Where there were differences in scoring, a consensus was reached. Extracted data was separately entered, reviewed and verified by two investigators (JLM, PLG) prior to analysis.

### Outcome measures

The primary outcome was mortality assessment at hospital discharge. Secondary outcomes were resolution of shock (or withdrawal of inotropes) at 7 to 28 days and corticotrophin responsiveness, secondary infections and non-infective (gastro-intestinal bleeding and new-onset hyperglycemia) complications.

### Definitions

Severe sepsis and septic shock were defined after the 1992 American College of Chest Physicians and Society of Critical Care Medicine Consensus Conference [19]. Pre-1992 studies were reviewed to establish consistency with this definition. Secondary infections were defined generally as a positive culture from a normally sterile site. The time span of the studies suggested that definitions for secondary infections would be subject to revision; for example, the use of quantitative cultures in more recent calendar years [20]. Shock resolution was defined as a stable hemodynamic state for a period of 24 hours or more after weaning of vasopressor support. Corticosteroid dose was converted into hydrocortisone equivalents (mg) and expressed as total maximum realizable dose [20] accounting for total time of exposure (therapeutic dose-time and tapering). Where patient corticosteroid dose-time schedule was unavailable due to death and/or reporting, we used median survival time from the published Kaplan-Meier curve.

### Statistical analysis

The effect of corticosteroids compared with control on mortality; the proportion of patients experiencing shock-resolution at defined times; and infective and non-infective complications were assessed using Bayesian random-effects models [21], via WinBUGS software [22] using three simultaneous runs of the program with disparate starting values. The first 10,000 iterations were discarded and results were reported as the posterior median odds ratio (OR) with 95% credible intervals (CrI) on the basis of a further 100,000 iterations. As argued previously [20], the hazard ratio would have been the preferred metric for mortality effect due to varying event times. However, due to the variability in intra-trial reporting, this was not feasible. As the hazard ratio may be approximated from the OR [23], we chose the OR as an appropriate metric [24]. Bayesian parameter estimates, as opposed to frequentist, are probability distributions and hence there is no contradiction in computing both (i) a (median) point estimate and CrI and (ii) the posterior probability (*P*) that, say, the OR is more than 1 [25,26]. That is, "Bayesian methodology also allows us to make statements about the probability that the ORs are greater than 1 in cases in which the associated 95% CrI includes 1" [27]. A probability of 50% suggests a null effect, while *P *of at least 90% signifies harm and *P *less than 10% indicates benefit for the mortality, infective and non-infective endpoints and vice versa for the shock reversal endpoint [28]. Analysis was undertaken by stratifying between 'high-dose' and 'low-dose' corticosteroid therapy, as in Annane and colleagues [6] and after the categorization of daily treatment doses of hydrocortisone by Marik (high-dose corticosteroid >1,000 mg per day) [29].

Bayesian meta-regression [21] was used to determine the relation between log odds mortality and (i) average patient age and (ii) control-arm risk, as log-odds mortality [17,24]. The slope (β) with 95% CrI and the probability that β ≥ 0 (*P*_β_) were presented. Heterogeneity was presented as the standard deviation, *τ*, between studies [30]; *τ *close to 0 indicates little heterogeneity, *τ *= 0.5 indicates moderate and *τ *> 1 reflects substantial heterogeneity [18].

For heuristic purposes we separately estimated: (i) pooled estimates with the Schumer [31] and Cooperative Study Group (CSG) [32] studies removed in a sensitivity analysis due to previous identification of the former as a potential outlier [7] and the remoteness of the latter 1963 trial from current therapeutic regimens; (ii) certain parameters of clinical import in the risk difference metric [21], albeit this metric may suffer from potential bias with varying time to event [24]; (iii) the mortality OR and probability (*P*) that the OR was 1 or more in the predictive distribution (that is, in the next 'new' study); (iv) the mortality OR for hypothesized studies of size 2,000 and 4,000 patients; (v) the Bayesian predictive *P*-value that the CORTICUS trial [8] was inconsistent with the other trials of the low-dose corticosteroid group; that is, the CORTICUS study was omitted from analysis (leaving *n *= 7 trials) and a replicate study of the same size as the CORTICUS study was drawn, with a replicate baseline, and a new treatment effect was established based upon the predictive distribution. A Bayesian predictive P-value was subsequently obtained, expressing the probability that the future study would be as 'extreme' as that observed.

Publication and the associated phenomenon of small-study bias were addressed using the approach of Peters and colleagues [33] via contour-enhanced funnel plots; formal quantitative testing for small-study-bias was performed using the approach of Harbord and colleagues [34], which has effective properties in the presence of appreciable heterogeneity. Implementation was via the R package 'meta' [35] and user-written routines.

## Results

**Figure 1:**
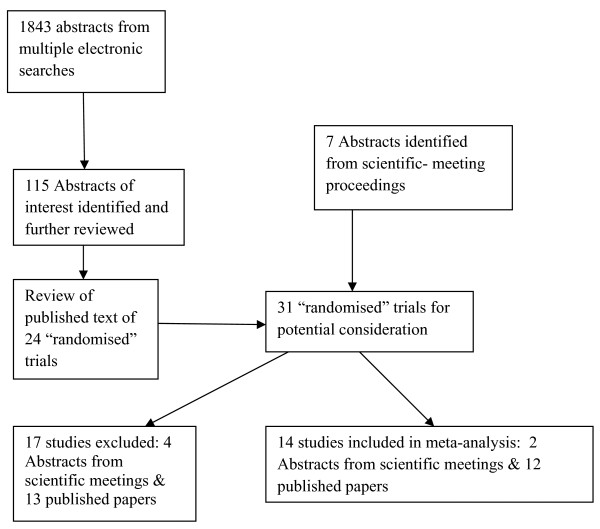
**Flowchart for identification of studies on corticosteroids in severe sepsis and septic shock; number of trials evaluated at each stage of the systematic review**.

Using multiple electronic searches, 1,843 abstracts of published papers were identified (including duplicates). A review of these abstracts (JLM, PLG) identified 115 papers of potential interest including review papers. The published text of 31 'randomized' clinical trials, including seven abstracts from proceedings of scientific meetings, were further reviewed (JLM, PLG, AB): two were excluded on the basis of reporting from previous trials, one reported no mortality outcome data and one used pseudo-randomization. A further 13 studies, including four abstracts-only were excluded for reasons given in Table 1. The final cohort was of 14 trials [8,31,32,36-46], including two abstracts from the reports of scientific meetings; 11 of the studies had been considered by previous meta-analyses [4-6,10] and the three remaining studies [8,42,44] were post-2004, the publication date of the two comparator meta-analyses [6,7] (Figure 1 and Table 2). The trial patient size varied from 28 [44] to 499 [8] and the total number of patients was 1,991, of mean age 55 years and 66% male. Total corticosteroid dosage in the high-dose cohort ranged from 7,000 to 42,000 hydrocortisone-equivalent mg over one to three days, whereas in the low-dose cohort, dosage was 856 to 2,175 hydrocortisone-equivalent mg over 2 to 10 days treatment with 0 to 14 days of tapering (Table 3). Average high- and low-dose control arm mortalities were 47% and 54%, respectively. Further characteristics of the trials are given in Tables 2 and 3.

**Table 1:** Study exclusions

Study	Year published	Reason for exclusion
Wagner and colleagues [78]	1955	Description of pneumonia therapy only with no severity stratification. Allocation by 'history number'
Thompson and colleagues [79]	1976	Abstract; nine of 60 patients with cardiogenic shock; no subset analyses. Post-randomization exclusion of 4 patients
Lucas and Ledgerwood [80]	1984	Open-label study; pseudo-randomization by hospital number
VASSCS [81]	1987	Predominantly sepsis patients with no subgroup of shocked patients. No timing of fluid bolus with respect to reported hypotension
Schattner and colleagues [82]	1997	pseudo-randomization of patients with 'early sepsis'
Keh and colleagues [60]	2003	Cross-over placebo study in septic shock
Confalonieri and colleagues [83]	2005	Community acquired pneumonia study; no subset analyses for shocked patients
Rinaldi and colleagues [84]	2006	Post randomization exclusion of 15 patients; 3 with septic shock
Huh and colleagues [85]	2006	Abstract; two hydrocortisone arms; no concurrent placebo arm reported
Loisa and colleagues [86]	2007	Two hydrocortisone arms; no concurrent placebo group
Nawab and colleagues [87]	2007	Abstract; severe community acquired pneumonia, no subset analysis; outcomes today-7 only
Cicarelli and colleagues [88]	2007	Unspecified post-randomization exclusion of 'all patients who progressed to refractory septic shock'
Kurugundla and colleagues [89]	2008	Abstract; ICU outcomes reported only

VASSCS, Veterans Administration Systemic Sepsis Cooperative Study.

**Table 2:** Final study cohort

Study	Year published	Year completed	Trial origin	Trial	Reported as Paper#/abstract	Design	Allocation concealment	Effect and sample size calculation	Early stopping	Sepsis/shock description	Predominant patient type	Primary outcome
Cooperative Study Group [32]	1963	NA	USA	Multicenter	Paper	Double-blind	Yes	No	No	'Life threatening infections'	Medical	Hospital mortality
Klastersky and colleagues [41]	1971	1970	Belgium	Singlecenter	Paper	Double-blind	Yes	No	No	'Life threatening infections'	Cancer	30-day mortality
Schumer [31]	1976	1975	USA	Singlecenter	Paper	Double-blind	NA	No	No	Septic history, falling blood pressure and positive blood cultures	Surgical	28-day mortality
Sprung and colleagues [43]	1984	1982	USA	Two-centers	Paper	Open-label	Yes	No	No	SBP < 90 mmHg, decreased organ perfusion hypotension despite fluid infusion, and bacteraemia or identified infection source	Medical	Hospital mortality
Bone and colleagues [38]	1987	1985	USA	Multicenter	Paper	Double-blind	Yes	No	No	Evidence of infection, fever/hypothermia tachypnoea, inadequate organ perfusion/dysfunction, SBP < 90 mmHg/decrease 40 mmHg	Mixed	SS development ≤14 days post admission; reversal SS ≤14 days; death ≤14 days
Luce and colleagues [46]	1988	1986	USA	Singlecenter	Paper	Double-blind	Yes	Yes	Yes	Fever/hypothermia, SBP < 90 mmHg, blood culture or body-fluid positive	Mixed	ARDS development, Hospital mortality
Bollaert and colleagues [37]	1998	NA	France	Singlecenter	Paper	Double-blind	Yes	Yes	Yes	ACCP/SCCM criteria	Mixed	Shock-reversal
Briegel and colleagues [39]	1999	1996	Germany	Singlecenter	Paper	Double-blind	Yes	Yes	No	ACCP/SCCM criteria	Mixed	Shock-reversal
Chawla and colleagues [47]	1999	NA	USA	Singlecenter	Abstract	Double-blind	NA	NA	NA	NA	NA	Shock-reversal
Yildiz and colleagues [45]	2002	1999	Turkey	Singlecenter	Paper	Double-blind	No	No	No	ACCP/SCCM criteria	Medical	28-day mortality
Annane and colleagues [36]	2002	1999	France	Multicenter	Paper	Double-blind	Yes	Yes	No	Documented evidence of infection; fever/hypothermia; SBP < 90 mmHg, despite fluid and vasopressors; decreased organ perfusion; mechanical ventilation	Mixed	28-day survival distribution in corticorophin non-responders
Tandan and colleagues [44]	2005	NA	India	Singlecenter	Abstract	Double-blind	NA	NA	NA	NA		28-day mortality
Oppert and colleagues [ 42]	2005	NA	Germany	Singlecenter	Paper	Double-blind	Yes	NA	NA	Tachycardia; Fever/hypothermia; Positive culture; SBP < 90 mmHg with CVP ≥10 mmHg,; vasopressors	Medical	Time to vasopressor cessation
Sprung and colleagues [8]	2008	2005	Europe	Multicenter	Paper	Double-blind	Yes	Yes	Yes	Clinical evidence of infection; evidence of systemic response to infection; shock (within 72 hours), SBP <90 mmHg despite fluid infusion or vasporessors; inadequate organ perfusion/dysfunction	Mixed	28-day mortality in corticorophin non-responders

#Paper, full publication as a journal paper; ACCP, American College of Chest Physicians; ARDS, Acute Respiratory Distress Syndrome; CVP, central venous pressure; NA, not-available; SBP, systolic blood pressure; SCCM, Society of Critical Care Medicine; SS, septic shock.

**Table 3:** Trial characteristics

Author	Quality score	Total *N*	Vasopressors atenrollment	Etomidate used	Steroid type	Steroid-days before tapering	Tapering days	Hydrocortisone equivalent (mg)	Age Steroid	Age Placebo	%males Steroid	%males Placebo
Cooperative Study Group [32]	7	194	NA	NA	Hydrocortisone	1	2-6	1050	NA	NA	70.8	64.3
Klastersky and colleagues [41]	7.5	85	NA	NA	Betamethasone	3	None	7000	NA	NA	60.1	46.2
Schumer [31]	6	172	NA	NA	Methyl-prednisolone	Bolus-day1	None	8050	49	51	NA	NA
					Dexamethasone	Repeat unspecified	None					
Sprung and colleagues [43]	8.5	59	Yes (93%)	NA	Methyl-prednisolone	Bolus-day1	None	18884	56.5	48	83.7	62.5
					Dexamethasone	Repeat in 74%						
Bone and colleagues [38]	10	382	NA	NA	Methyl-prednisolone	4 doses in 24 hours	None	42000	53	53.7	NA	NA
Luce and colleagues [46]	11	75	Yes (44%)	NA	Methyl-prednisolone	4 doses in 24 hours	None	42000	50	53	68.4	83.8
Bollaert and colleagues [37]	13.5	41	Yes	NA	Hydrocortisone	5	6	2175	58.7	56.8	68.2	63.2
Briegel and colleagues [39]	13.5	40	Yes	NA	Hydrocortisone	3	3-14	2126	47	51	45	60
Chawla and colleagues [47]	NA	44	Yes	NA	Hydrocortisone	3	4	1350	NA	NA	NA	NA
Yildiz and colleagues [45]	9	40	NA	NA	Prednisolone	10	None	300	57.8	56.5	65	55
Annane and colleagues [36]	14.5	291	Yes	Yes	Hydrocortisone	7	None	1400	62	60	64	69.8
Tandan and colleagues [44]	NA	28	NA	NA	NA	NA	NA	NA	NA	NA	NA	NA
Oppert and colleagues [42]	12.5	41	Yes	Yes	Hydrocortisone	2-3	2-5	856	59	47	72.2	82.6
Sprung and colleagues [8]	14.5	499	Yes (98.5%)	Yes	Hydrocortisone	5	6	1800	63	63	66.1	67.8

NA, not available.

The primary outcome of hospital mortality was available in six studies [8,32,36,39,43,46]; the other studies had recorded 28- or 30-day mortality and one study recorded 14-day mortality (Table 2). Sepsis and shock definitions of trials completed before 1992 [31,32,38,41,43,46] were generally consistent with definitions of the American College of Chest Physicians and Society of Critical Care Medicine Consensus Conference on sepsis and organ failure, albeit the two trials published in 1971 [41] and 1963 [32] used 'life threatening infections' as a criteria (Table 2). Of interest, trials before 1998 were predominantly reported from the USA; after 1997, they were from European and other non-USA sites. Trial patient data by outcomes (hospital mortality; shock-reversal; corticotrophin-responsiveness; shock reversal by corticotrophin-responsiveness; and secondary complications, as infectious, gastro-intestinal bleeding and new-onset hyperglycemia) are shown in Table 4.

**Table 4:** Trial patient data by outcome

Author	Mortality	Mortality	Shock-reversal	Shock-reversal	Corticotrophin	Corticotrophin	Shock-reversal	Shock-reversal	Shock-reversal	Shock-reversal	Superinfection	Superinfection	GIS bleed	GIS bleed	New	New
	Hospital	Hospital	days(7-28)	days(7-28)	responders	responders	responders	responders	non-responders	non-responders					hyperglycaemia	hyperglycaemia
	*n*/total	*n*/total	*n*/total	*n*/total			Days (7-28)	Days (7-28)	Days (7-28)	Days (7-28)						
	Steroid	Placebo	Steroid	Placebo	Steroid	Placebo	Steroid	Placebo	Steroid	Placebo	Steroid	Placebo	Steroid	Placebo	Steroid	Placebo
Cooperative StudyGroup [32]	54/96	32/98	NA	NA							3/96	3/99	4/96	0/98	NA	NA
Klastersky and colleagues [41]	24/66	18/39	NA	NA							11/46	6/39	NA	NA	NA	NA
Schumer [31]	9/86	33/86	NA	NA									2/86	1/86	1/86	1/86
Sprung and colleagues [43]	33/43	11/16	25/43	6/16							11/43	1/16	1/43	2/32	4/45	0/16
Bone and colleagues [38]	65/191	48/190	85/130	83/114							29/152	30/147	NA	NA	NA	NA
Luce and colleagues [46]	22/38	20/37	NA	NA							3/38	4/37	18/38	16/37	16/38	15/37
Bollaert and colleagues [37]^##^	7/22	12/19	15/22	4/23	18/22	11/19	12/18	2/11	3/4	2/8	7/22	9/19	1/22	3/19	3/22	3/19
Briegel and colleagues [39]	5/20	6/20	18/20	16/20	NA	NA	NA	NA	NA	NA	10/20	7/20	1/20	0/20	NA	NA
Chawla and colleagues [47]^#^	6/23	10/21	16/23	7/21	NA	NA	NA	NA	NA	NA	NA	NA	NA	NA	NA	NA
Yildiz and colleagues [45]	8/20	12/20	NA	NA	15/20	11/20	NA	NA	NA	NA	0/20	1/20	NA	NA	0/20	0/20
Annane and colleagues [36]	95/160	103/150	60/151	40/149	36/150	34/149	18/36	18/34	65/114	46/115	22/150	27/150	11/150	8/149	NA	NA
Tandan and colleagues [44]	11/14	13/14	5/14	3/14	NA	NA	NA	NA	NA	NA	NA	NA	NA	NA	NA	NA
Oppert and colleagues [42]	7/18	11/23	13/18	18/23	6/18	9/23	NA	NA	NA	NA	NA	NA	NA	NA	NA	NA
Sprung and colleagues [8]	111/251	100/245	200/251	184/248	118/243	136/244	100/118	104/136	98/125	76/108	78/234	61/132	15/234	13/232	186/234	161/232

#, mortality statistics for Chawla and colleagues [47] were abstracted from the Annane and colleagues meta-analysis[6]. ##, data for hyperglycaemia for Bollaert and colleagues [37] was abstracted from the Annane and colleagues meta-analysis[6]. GIS, gastrointestinal; NA, not available.

### Mortality outcome

**Figure 2:**
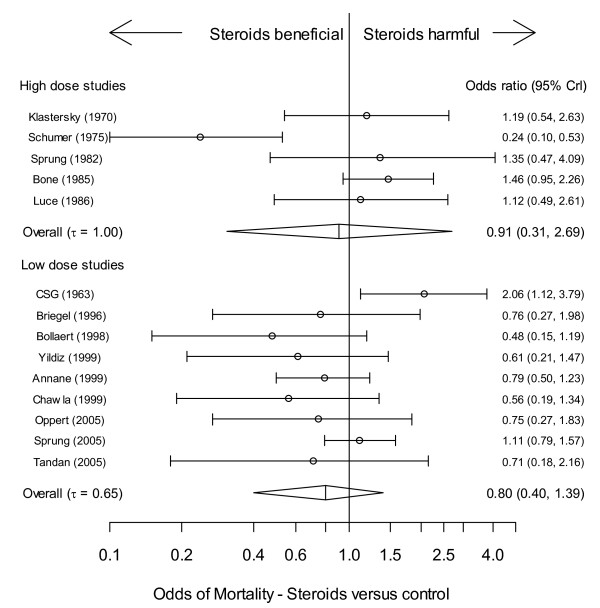
**Corticosteroid mortality effect (OR), stratified by high (upper panel) or low (lower panel) dose steroid regimen; forest plot representation of the effect**. The vertical straight line denotes null effect (odds ratio (OR) = 1). The individual points denote the OR for each study and the lines on either side the 95% Bayesian credible intervals (CrI).

Neither the low-dose nor high-dose cohort showed a significant steroid treatment effect for the mortality OR, although there was modest evidence of benefit in the low-dose cohort (*P *= 20.4%) (Table 5 and Figure 2). The odds of mortality (four studies [8,36,42,45]), for both corticotrophin responders and non-responders was not significantly different compared with control (Table 5).

**Table 5:** Outcome effect estimates

Outcome		N	OR (95%CrI)	*P *(%)	τ (95%CrI)	β (95%CrI)	***P***_**β**_**(%)**
**Mortality**							
High dose		5	0.912 (0.313 to 1.253)	42.0	1.00 (0.42 to 1.89)		
High dose excluding Schumer [31]		4	1.406 (0.727 to 2.614)	89.3	0.25 (0.01 to 1.40)		
Low dose		9	0.796 (0.396 to 1.386)	20.4	0.65 (0.23 to 1.44)		
Low dose excluding CSG [32]		8	0.706 (0.371 to 1.096)	5.8	0.39 (0.04 to 1.15)		
Corticotrophin responders*		4	0.882 (0.285 to 2.073)	36.4	0.49 (0.02 to 1.78)		
Corticotrophin non-responders*		4	0.831 (0.334 to 1.971)	28.0	0.43 (0.02 to 1.69)		
**Shock-reversal**							
High dose		2	1.078 (0.227 to 6.311)	54.9	1.39 (0.06 to 1.93)		
Low dose		7	1.999 (1.069 to 4.55)	98.2	0.57 (0.04 to 1.62)		
Corticotrophin responders*		3	1.830 (0.499 to 7.845)	86.7	0.87 (0.05 to -1.92)		
Corticotrophin non-responders*		3	1.845 (0.637 to 7.267)	91.9	0.55 (0.02 to 1.86)		
**Meta-regression (log odds mortality)**							
Average age	High dose	4	0.777 (0.285 to 2.426)	27.3	0.72 (0.04 to 1.87)	0.60 (-0.23 to 1.51)	94.52
	Excl Schumer [31]	3	1.390 (0.399 to 4.872)	77.0	0.66 (0.03 to 1.90)	0.10 (-1.57 to 1.74)	58.05
	Low dose	6	0.658 (0.334 to 1.223)	7.6	0.36 (0.02 to 1.51)	0.05 (-0.10 to 0.18)	80.53
Underlying risk	High dose	5	0.943 (0.292 to 3.049)	45.4	1.14 (0.46 to 1.49)	0.23 (-1.71 to 2.58)	60.98
	Excl Schumer [31]	4	1.372 (0.596 to 3.249)	82.9	0.38 (0.01 to 1.74)	-0.09 (-1.31 to 1.42)	41.47
	Low dose	9	0.752 (0.389 to 1.291)	14.5	0.57 (0.17 to 1.37)	-0.49 (-1.14 to 0.27)	7.80
	Excl CSG [32]	8	0.676 (0.347 to 1.076)	4.9	0.40 (0.03 to 1.23)	-0.28 (-0.88 to 0.50)	19.08
**Odds of the following complications (corticosteroids versus control)**							
Superinfection	High dose	4	1.127 (0.364 to 3.924)	62.2	0.55 (0.02 to 2.85)		
	Low dose	6	0.955 (0.388 to 1.749)	43.6	0.46 (0.03 to 1.62)		
GI bleeding	High dose	3	0.824 (0.167 to 3.186)	37.3	0.74 (0.03 to 1.90)		
	Low dose	5	1.103 (0.379 to 3.031)	59.6	0.58 (0.02 to 1.84)		
Hyperglycemia	High dose	3	1.012 (0.244 to 4.266)	50.8	0.64 (0.03 to 1.88)		
	Low dose	3	1.430 (0.155 to 3.985)	57.4	0.87 (0.05 to 1.93)		

*all studies were low dose; CI, confidence interval; CSG, Cooperative Study Group; GI, gastro-intestinal; N, number of studies reporting data for that endpoint; NA, not applicable; OR, odds ratio. Excl Sch = Excluding Schumer [31]; Excl CSG = Excluding CSG [32]

**Figure 3:**
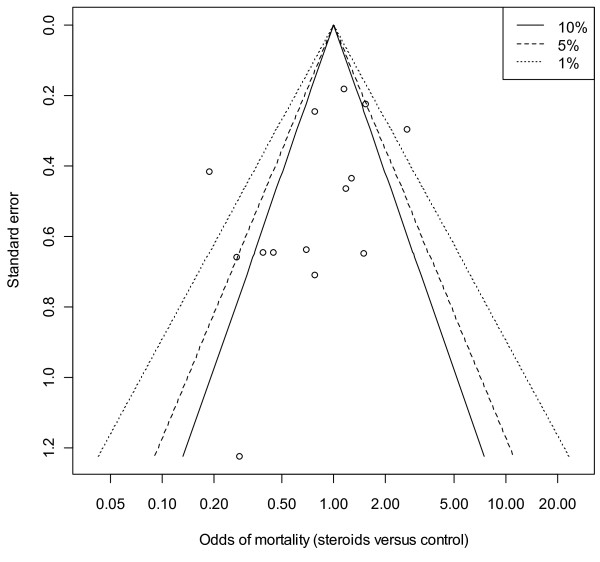
**Contour-enhanced funnel plot of mortality odds versus standard error for all trials (*n *= 14)**. Vertical axis, standard error; horizontal axis, mortality odds (log scale). The 'contours', based upon a two-sided *P *value, are the conventional levels (not 'pseudo' confidence intervals) of statistical significance (<0.01, <0.05, <0.1) for the primary studies and are independent of the pooled estimate (if the pooled estimate is biased, the contours are not affected) [33].

**Figure 4:**
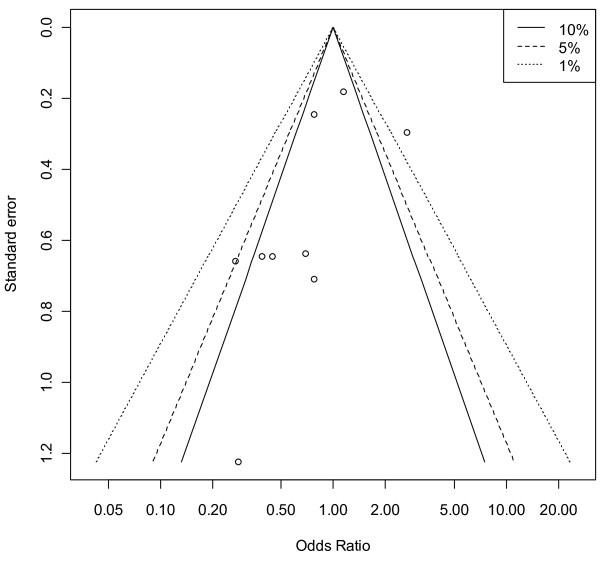
**Contour-enhanced funnel plot of mortality odds versus standard error for low-dose corticosteroid trials (*n *= 9)**. Vertical axis, standard error; horizontal axis, mortality odds (log scale). The 'contours', based upon a two-sided *P *value, are the conventional levels (not 'pseudo' confidence intervals) of statistical significance (<0.01, <0.05, <0.1) for the primary studies and are independent of the pooled estimate (if the pooled estimate is biased, the contours are not affected) [33]

A contour-enhanced funnel plot showed no obvious asymmetry in terms of a lack of small studies with a null or adverse steroid effect (Figure 3), this was not rejected (at the 0.1 level) using the quantitative approach of Harbord and colleagues [34] (*P *= 0.146). The low-dose studies showed a degree of asymmetry of the contour-enhanced funnel plot (Figure 4), but the quantitative estimate did not confirm this (*P *= 0.113).

### Shock reversal

**Figure 5:**
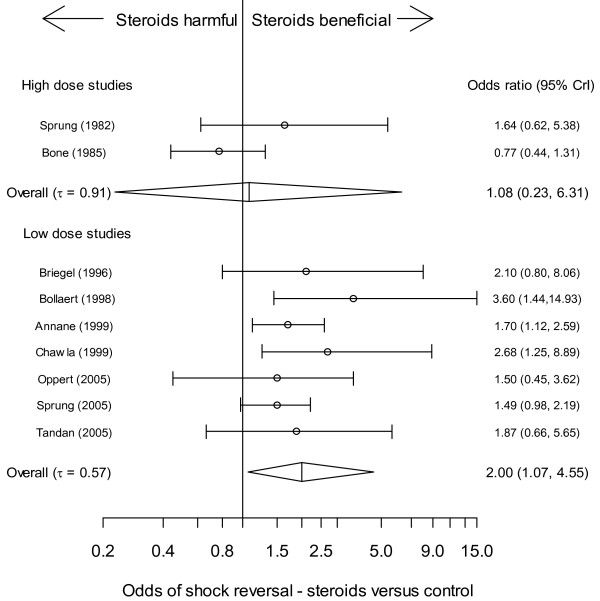
**Corticosteroid shock-reversal effect (OR), stratified by high (upper panel) or low (lower panel) dose steroid regimen; forest plot representation of the effect**. The vertical straight line denotes null effect (odds ratio (OR) = 1). The individual points denote the OR for each study and the lines on either side the 95% Bayesian credible intervals (CrI).

Median vasopressor time (six studies [8,36,37,39,42,47]) ranged from 2 to 7 days for steroid-treated patients and 5 to 13 days for placebo. With respect to the number of patients experiencing shock reversal, there was no clear steroid treatment-effect (overall OR included 1) for high-dose studies (*n *= 2). However, there was a high probability of benefit for the low-dose cohort; moderate heterogeneity being present (Figure 5 and Table 5). Odds of shock-reversal were not substantially different for corticotrophin non-responders or responders; however, both had a high probability of benefit (Table 5).

### Metaregression

Univariate metaregression of average age against log odds mortality yielded no significant effects although *P*_β _was high and the slope positive for both low- and high-dose cohorts. This indicated some evidence that, on average, older study participants had increased odds of mortality under steroid treatment versus control (Table 5). Similarly, although the metaregression of underlying control-arm risk against log odds mortality yielded no significant effects, for the low-dose cohort, *P*_β _was small and the slope negative, indicating a high probability that as the underlying risk of mortality increased the log odds mortality under steroid treatment decreased (Table 5). The removal of the CSG study [32] attenuated the negative slope of the line. In the risk difference metric, the intersection of the (meta)regression line with the line of null effect ('cross-over' point) occurred for age at 62 years and for control-arm mortality at 44%.

### Complications of therapy

The complications of therapy were secondary infections, gastro-intestinal bleeding and steroid-induced hyperglycemia. No overall or low- or high-dose effects were demonstrated for any of the pooled endpoints (Table 5).

### Heuristics

The considerable heterogeneity of the high-dose cohort (τ = 1.00, 95%CrI = 0.42 to 1.89) was diminished by the removal from analysis of the Schumer study [31] (Table 5), an effect previously noted by Minneci and colleagues [7]. This also lead to a high probability of harm in the high-dose studies (*P *= 89.3%) although the CrI for the OR still included 1. Removal of the CSG study [32] from analysis resulted in reduced heterogeneity among the low-dose studies (Table 5) and a high probability of benefit in the low-dose studies (*P *= 5.8%) although the CrI for the overall OR just included 1.

In the risk difference metric the absolute risk difference (treatment versus control) for nine trials in the low-dose cohort was -0.047(95%CrI = -0.197 to 0.077; *P*(RD > 0) = 21.9%); and for eight trials (CSG trial excluded [32]) was -0.072 (95%CrI = -0.202 to 0.018; *P*(RD > 0) = 5.3%), similar to the 6.6% reported by Annane and colleagues [48]. The mortality OR in the predictive distribution (from eight trials) was 0.703 (95%CrI = 0.156 to 2.198; *P*(OR > 1) = 19.9%). For hypothesized studies of size 2,000 and 4,000 patients, the mortality ORs were predicted to be 0.724 (95%CrI = 0.184 to 2.108) and 0.726 (95%CrI = 0.184 to 2.096), respectively. The Bayesian predictive P-value, reflecting the inconsistency of the CORTICUS study [8] with the remaining trials (*n *= 7; CSG trial excluded [32]) was 0.074.

## Discussion

Despite the disappointment of the CORTICUS [8] trial, our review suggests a modest to high probability (80% to 98%) of efficacy for low-dose steroids with respect to both mortality and shock reversal; the mortality effect being risk-related (Table 5). These probabilities are to be interpreted in the context of CrI spanning the null for all estimates (see Statistical analysis, above). We found no strong evidence for the determinacy of ACTH responsiveness nor complications of corticosteroid therapy. This being said, it is of interest to note the admonitory impact of the CORTICUS study on recent summary statements of sepsis management [2,3,13,29,49]. Consistent with previous meta-analyses [6,7] we found null or adverse effects of high-dose steroids; the probability of therapeutic complications being low (Table 5).

The use of prolonged low-dose corticosteroid was justified in the landmark Annane and colleagues trial on the basis that "severe sepsis may be associated with relative adrenal insufficiency or systemic inflammation-induced glucocorticoid receptor resistance..." [36]. Apropos of this statement, it is instructive to note that the primary aim of the CORTICUS study was 28-day mortality in patients not responding to corticotrophin [8]. A recent review of corticosteroid insufficiency in the critically ill has suggested that in states where such insufficiency [50] is located "within the tissue itself...the adrenal gland function could be normal... it would be impossible to diagnose this state on the basis of serum or even tissue levels of glucocorticoids...[and]...treatment would require supraphysiological levels of glucocorticoids" [51]. The inability in the current meta-analysis to demonstrate treatment efficacy with respect to mortality and shock-reversal based upon corticotrophin responsiveness is in agreement with Minneci and colleagues [7] and suggests both that tests of the latter to direct treatment regimens are misplaced and that the notion of adrenal insufficiency in severe sepsis and septic shock is problematic [52]; a "...hardly definable disease entity or syndrome..." [53].

Of the seven trials reporting shock-reversal [8,36,37,39,40,42,44], time to the latter end-point was the primary study end-point in three [37,39,42]. All published studies used time-to-event analysis based upon conventional Kaplan-Meier estimates, censoring those who died and/or those in whom vasopressor therapy could not be withdrawn at time of assessment. However, such analyses are problematic, because they ignore the competing risk of those who died and/or those in whom vasopressor therapy could not be withdrawn. In the presence of competing risks Kaplan-Meier estimates cannot be interpreted as probabilities [54,55]. Under the conditions of competing risks, the probability of an event is more appropriately estimated by the cumulative incidence function, which, for the particular event of interest, is a function of the hazards of all the competing events and not solely of the hazard of the event to which it refers. Hypothesis tests for the cumulative incidence function do not necessarily equate with the familiar log-rank test [56].

How then are we to understand these favourable effects of low-dose corticosteroids? Glucocorticoid action on inflammation [57], vascular reactivity [58] and interactions between corticosteroids and 'signalling pathways' [59] may explain the salutary effects in sepsis [60]; anti-inflammatory and coagulation effects would appear to be differentially dose dependent [61]. Low or stress doses of hydrocortisone, as currently prescribed, are not replacement or physiological doses; they generate plasma cortisol levels greater than 2,500 nmol/l, in excess of the usual upper limits (1,000 to 1,500 nmol/l) of patients in septic shock [42,60,62]. The presumed immune-modulation [63] of these prolonged low-dose regimens underpins the rationale of critical illness-related corticosteroid insufficiency [14,29]. This being said, the Annane and colleagues [36] trial used a fixed seven-day steroid course without tapering and claimed efficacy and no difference in the complication rates was evident between the high-and low-dose cohorts in both the current and Annane and colleagues' meta-analyses [6]. As mentioned in commentary [64], differences in control group mortalities of the Annane and colleagues [36] and CORTICUS [8] trials may explain differing outcomes on the basis of risk-related treatment effects. The latter were persuasively demonstrated in the current meta-analysis. The estimate of mortality risk at which low-dose corticosteroids began to exhibit a treatment effect, 44%, was clinically plausible given the range of control-arm mortalities of 30 to 93%. Such demonstration, using appropriate Bayesian methodology [17,24], represents a novel insight into critical care therapeutic efficacy.

### Critique of methodology

Our analytic approach was to consider the two treatment cohorts, high- and low-dose corticosteroid, separately; we did not produce an overall treatment effect on the basis that both the treatment intention and effective (daily) corticosteroid dose of the two cohorts were quite disparate. An alternate approach would have been to consider all trials (*n *= 14) with total hydrocortisone dose or calendar year as effect-moderators. In the absence of individual patient data, such analyses, with only 14 studies, have low power.

Secondary outcome analysis was beset by selection bias in reporting [65], as witnessed by study numbers in Table 5; parameter estimates may be biased under such circumstances. The study list addressing low-dose corticosteroid mortality efficacy (*n *= 9) included a single study [32] in 1963, the others being from the period 1996 to 2005 (Figure 2). Plausible estimates of current therapeutic efficacy would suggest analysis excluding the former study, the result of which was to reduce heterogeneity of the mortality effect by 40% and to reveal a probability of corticosteroid efficacy of 94.2% (Table 5). The single-investigator single-centre Schumer study, conducted over a prolonged eight-year period, has been previously subject to substantive critique [7] and recent cautions regarding extended recruitment time [66] and inference from single-center studies [67] merits its consideration as an outlier.

That the inclusion of the large but null-effect CORTICUS trial [8] in the current meta-analysis did not extinguish a probable treatment effect deserves comment. The impact of the single large trial is undoubted, but the evidence produced by such a trial may be "less reliable than its statistical analysis suggests" [68]. We adopted a random effects methodology [69] in the presence of moderate between study heterogeneity (τ, Table 5); under these conditions large studies may have little impact upon a meta-analysis [70] and there may be virtue in (clinical) heterogeneity [71]. The degree of asymmetry of the contour-enhanced funnel plot in the low-dose cohort (see Results, Mortality outcome, above) raises concerns about a random effects methodology [69], but there was no quantitative evidence of small-study effects (at the 0.1 level) and the number of studies was small. In the presence of sparse data and moderate heterogeneity (Table 5), the interpretation of funnel plot asymmetry is problematic [34,72] and exploration of the reasons for such heterogeneity is the preferred analytic focus [34].

With respect to the efficacy of corticosteroids in severe sepsis and septic shock, the divergent positions represented by the Annane and colleagues [36] and CORTICUS [8] trials remain unresolved. Two recent (calendar year 2009) updates [48,73] of previous meta-analyses [6,7] also merit comment. Both of the updated meta-analyses, using frequentist methodology, found efficacy of low-dose prolonged corticosteroids with respect to the mortality effect, Annane and colleagues [48] found a relative risk of 0.84 (95% confidence interval (CI) = 0.72 to 0.97; *P *= 0.02) and Minneci and colleagues [73] found an OR of 0.64 (95% CI = 0.45 to 0.93; *P *= 0.02), and shock reversal, the latter effect consistent with the estimates of the current study (Table 5). Study inclusions in these meta-analyses differed and were not the same as in our meta-analysis, which adopted a rigorous exclusion policy (Table 1). The frequentist meta-regression methods used by both meta-analyses [48,73] to estimate the risk-related treatment efficacy of steroids are problematic [17,24]. Although such methods may identify putative risk related treatment effects in meta-analyses they fail to allow for both regression to the mean (the difference between outcome and baseline being correlated with baseline) and the stochastic nature of the control rate (regression dilution bias). The stochastic characteristic of the control rate is also not addressed as the expected response in (ordinary) linear regression is conditional upon independent (fixed) variables and there is no inherent accounting for the random error in estimation of this control rate. Such problems are overcome by the use of Bayesian methods [17,24].

Both meta-analyses were judicious in their conclusions about treatment efficacy and this was reiterated by an accompanying editorial [74]. However, neither study was able to attend to this uncertainty in a tangible manner. This is precisely what our Bayesian analysis quantifies: what was the probability of treatment efficacy. For example, our analysis demonstrated that the probability of adverse mortality outcome with low-dose corticosteroids (outlier excluded) was 5.8% (Table 5). The omission of such a probability statement cannot be justified by an appeal to "the nominal *P *values for these outcomes were very close to 0.05...." [48]. We have previously cautioned the against interpretation of 95% CI (and associated frequentist *P *values) as probability statements [75]. Furthermore, neither meta-analysis reported exploration of estimates from a predictive distribution, which may be considered as a more appropriate future treatment summary than the mean effect [18]. Such a capacity recommends Bayesian methodology, although meta-analytic prediction intervals, which address the "...dispersion of the effect sizes..." are computable from a frequentist perspective [76]. With respect to reservations expressed regarding the status of the CORTICUS study [29,74], we found no compelling evidence (Bayesian predictive *P*-value 0.074) that this trial was inconsistent with the remaining (*n *= 7) trials.

Continued controversy and conventional wisdom [77] would appear to mandate the conduct of a large-(mega)-trial of this therapy in well-defined patient subsets; an absolute treatment effect of 7.2%, control arm risk of 54% and 90% power would suggest a total patient number of greater than 2,000. This being said our predictive estimates were unable to suggest efficacy for future 'large' trials, albeit the trial base from which these estimates were made was small.

## Conclusions

Although a null effect for mortality treatment efficacy of low-dose corticosteroid therapy in severe sepsis and septic shock could not be excluded, there appears to be credible evidence for shock reversal efficacy. Similarly, although a null effect was not excluded, advantageous effects of low-dose steroids had a high probability of dependence upon patient age and underlying risk. Low-dose steroid efficacy was not demonstrated in corticotrophin non-responders. Bayesian methods are apposite to express uncertainty in efficacy estimates from meta-analyses.

## Key messages

• The efficacy of corticosteroids in patients with severe sepsis and septic shock is uncertain despite recent meta-analytic reviews.

• Bayesian methods are apposite to express uncertainty in efficacy estimates from meta-analyses.

• The efficacy of low-dose corticosteroids had a high probability of dependence upon patient age and underlying risk; low-dose steroid efficacy was not demonstrated in corticotrophin non-responders.

• Bayesian meta-analytic predictive estimates were unable to suggest efficacy for future large trials.

• A null effect for mortality treatment efficacy of low-dose corticosteroid therapy in severe sepsis and septic shock could not be excluded.

## Abbreviations

ACTH: adreno-corticotrophin hormone; CI: confidence interval; CrI: credible intervals; CSG: Cooperative Study Group; OR: odds ratio; CORTICUS: Corticosteroid Therapy of Septic Shock ACCP: American College of Chest Physicians; SCCM: Society of Critical Care Medicine.

## Competing interests

The authors declare that they have no competing interests.

## Authors' contributions

The study was conceived by JLM, PLG and AB. SR constructed the search terms and conducted the electronic search. JLM, PLG and AB reviewed studies fulfilling inclusion criteria and pre-defined variables. JLM, and PLG conducted the quality assessment and statistical analysis. All authors contributed to the writing of the paper, critical review and final approval.

## Supplementary Material

Additional file 1 **Electronic search strategy**. Detailed search strategy of electronic databases Click here for file
