# Supplementary material for: Updating the evidence for the role of corticosteroids in severe sepsis and septic shock: a Bayesian meta-analytic perspective
Source: Crit Care. 2010 Jul 13;14(4):R134. doi: 10.1186/cc9182 (PMC2945102; doi:10.1186/cc9182)
Supplement: Additional file 1 — Electronic search strategy. Detailed search strategy of electronic databases [file cc9182-S1.DOC]

Search terms:

Search strategy developed to use with OVID Medline, OVID Embase and EBSCO Cinahl. Search strategy uses subject headings where possible and free text searching where there are no appropriate subject headings in the thesaurus. (sepsis/ or exp Shock, Septic/ or bacteremia/ or fungemia/ or sepsis.mp. or exp Pneumonia/ or pneumonia.mp or septicemia.mp. or "septic shock".mp.) AND (exp Hydrocortisone/ or corticosteroids.mp. or exp Adrenal Cortex Hormones/ or exp Steroids/ or hydrocortisone.mp. or steroid?.mp.) AND (limit to clinical trial, all or randomized controlled trial or controlled clinical trial or meta analysis or (randomised controlled trial$ or clinical trial$ or RCT$).mp.)
